# Urinary Biomarkers in Bladder Cancer: Where Do We Stand and Potential Role of Extracellular Vesicles

**DOI:** 10.3390/cancers12061400

**Published:** 2020-05-29

**Authors:** Manuel Castanheira de Oliveira, Hugo R. Caires, Maria J. Oliveira, Avelino Fraga, M. Helena Vasconcelos, Ricardo Ribeiro

**Affiliations:** 1 i3S—Instituto de Investigação e Inovação em Saúde, University of Porto, 4200-135 Porto, Portugal; hcaires@ipatimup.pt (H.R.C.); mariajo@ineb.up.pt (M.J.O.); avfraga@gmail.com (A.F.); hvasconcelos@ipatimup.pt (M.H.V.); 2 Tumor & Microenvironment Interactions Group, INEB - Institute of Biomedical Engineering, University of Porto, 4200-135 Porto, Portugal; 3 Department of Urology, Centro Hospitalar e Universitário do Porto, 4099-001 Porto, Portugal; 4 ICBAS-Institute of Biomedical Sciences Abel Salazar, University of Porto, 4050-313 Porto, Portugal; 5 Cancer Drug Resistance Group, IPATIMUP - Institute of Molecular Pathology and Immunology of the University of Porto, 4200-135 Porto, Portugal; 6 Department of Pathology, Faculty of Medicine, University of Porto, 4200-450 Porto, Portugal; 7 Department of Biological Sciences, FFUP—Faculty of Pharmacy, University of Porto, 4050-313 Porto, Portugal; 8 Laboratory of Genetics and Instituto de Saúde Ambiental, Faculdade de Medicina, University of Lisbon, 1649-028 Lisbon, Portugal; 9 Department of Clinical Pathology, Centro Hospitalar e Universitário de Coimbra, 3004-561 Coimbra, Portugal

**Keywords:** extracellular vesicles, microvesicles, bladder cancer, urothelial cancer, biomarkers, liquid biopsy

## Abstract

Extracellular vesicles (EVs) are small membrane vesicles released by all cells and involved in intercellular communication. Importantly, EVs cargo includes nucleic acids, lipids, and proteins constantly transferred between different cell types, contributing to autocrine and paracrine signaling. In recent years, they have been shown to play vital roles, not only in normal biological functions, but also in pathological conditions, such as cancer. In the multistep process of cancer progression, EVs act at different levels, from stimulation of neoplastic transformation, proliferation, promotion of angiogenesis, migration, invasion, and formation of metastatic niches in distant organs, to immune escape and therapy resistance. Moreover, as products of their parental cells, reflecting their genetic signatures and phenotypes, EVs hold great promise as diagnostic and prognostic biomarkers. Importantly, their potential to overcome the current limitations or the present diagnostic procedures has created interest in bladder cancer (BCa). Indeed, cystoscopy is an invasive and costly technique, whereas cytology has poor sensitivity for early staged and low-grade disease. Several urine-based biomarkers for BCa were found to overcome these limitations. Here, we review their potential advantages and downfalls. In addition, recent literature on the potential of EVs to improve BCa management was reviewed and discussed.

## 1. Introduction: Bladder Cancer and Disease Management

Urological tumors represent approximately 25% of all human cancers [1]. Bladder cancer (BCa) is the 10th most common, and the 9th cause of death by malignancy worldwide [2]. Aging, ethnicity, and male gender are considered non-modifiable risk factors [2,3,4], but most tumors are derived from acquired environmental exposure to carcinogenic substances. Cigarette smoking is considered the main risk factor [5,6], with an estimated causal association for half of BCa in both genders [7,8,9], whereas occupational exposure accounts for 10–20% of all cases [10]. The worldwide incidence of BCa seems to reflect areas with higher exposure to risk factors [10], which explains why developed countries have a larger number of diagnosed cases [2,10]. As an example, *Schistosoma haematobium* infection, another known risk factor, is more prevalent in northern and sub-Saharan African countries, where there is a relatively higher incidence of BCa [10]. In addition, differences in healthcare systems also account for disparity of incidence rates, being better resources associated to an easier and faster diagnosis. 

Urothelial cancer originates in the epithelial cells of the urothelium, extending from the renal pelvis to the urethra [11,12,13]. The majority of these tumors are located in the bladder, accounting for 90–95% of cases, while 5–10% are located in the upper urinary tract (UUT) [14,15,16,17,18]. Tumor extension is classified according to the TNM (Tumor-Node-Metastasis) staging system. At diagnosis, approximately 75–80% of bladder tumors are non-muscle invasive (NMIBC), which includes mucosa (for stages Ta and Cis) and lamina propria (T1 stage) confined disease, while 20–25% are muscle-invasive (MIBC), when invading the muscle layer and beyond (T2–T4 stages) [1,4,14].

Although clinical presentation may be suggestive, the gold standard diagnostic procedures are cystoscopy and urinary cytology [19,20,21,22,23,24]. Nevertheless, cystoscopy is an invasive, operator-dependent procedure, with low sensitivity for small papillary or Cis tumors, which, if underdiagnosed and untreated, progress to muscle-invasive disease in approximately half of the patients [19,20,21,22,23,24]. The sensitivity and specificity of white light cystoscopy is 71% (95% CI: 0.49–0.93%) and 72% (95% CI: 47–96%), respectively [24]. However, due to its invasiveness, it is frequently associated with side effects such as dysuria (50%), hematuria (19%), or urinary tract infection (3%) [25,26].

As for urinary cytology, it has high diagnostic accuracy for high grade lesions and Cis, with a sensitivity of 80–90% and specificity between 98% and 100% [27]. However, it exhibits low sensitivity for low grade lesions, between 4% and 31% [28,29,30,31,32,33], and high rate of false positives, due to benign or inflammatory conditions produced by chemo or radiation therapy [34,35]. To overcome these limitations, several urinary biomarkers were developed and are currently commercially available. Compared to cytology, they have higher sensitivity but lower specificity and are, unfortunately, less useful in low risk BCa [36,37,38]. Therefore, consensus among the different international societies on these biomarkers still do not recommend them as replacements of cytology in the current clinical practice [36,37,38].

The standard therapy for NMIBC is trans-urethral resection of the bladder (TURB), with both diagnostic and therapeutic purposes, complemented or not by intravesical adjuvant treatment [39,40]. However, even after complete endoscopic resection, there is a high recurrence rate, around 50–70%, and 10–30% will progress to MIBC [39,40]. This feature of BCa natural history elicits the need for a regular follow-up with cystoscopy and cytology at every 3 months interval, generally accompanied by repeated treatments due to recurrence, and which frequently result in high morbidity and economic burden [1,41,42].

Driven by the invasiveness and morbidity of cystoscopy, the lack of acceptable sensitivity of urinary cytology and of specificity of the commercially available urinary diagnostic biomarkers, urge the need for extensive research on the identification of novel and more effective biomarkers, to implement better tools for diagnosis, follow-up, and screening of at risk populations [1,29,34,42,43,44].

Extracellular vesicles (EVs) are small membrane vesicles which have emerged as a source of biomarkers in bladder cancer [45]. Their detection in liquid biopsies is feasible, due to their presence and stability in most human fluids, and may serve as biomarkers in bladder cancer early detection as they present similar cargo to their donor cancer cells [46]. Additionally, they have some advantages as a source of biomarkers since they are more abundant in liquid biopsies compared to circulating tumor cells (CTCs), protect their cargo against degradation and may carry molecular signatures associated with specific phenotypes [47,48,49]. The present review focus on the status of urinary biomarkers in diagnosis and follow-up of bladder cancer, pinpointing the emerging potential role of urinary EVs on bladder cancer diagnosis and management.

## 2. Liquid Biopsy as a Source of Biomarkers for Bladder Cancer

The ideal biomarker would be cost-effective, objective, fast to process, and easy to interpret, with high sensitivity and specificity [43,50,51,52,53,54]. For urothelial cancer biomarkers, four goals have been proposed to be accomplished: (i) reduce the need for frequent invasive procedures; (ii) exclude recurrence; (iii) detect progression towards invasive disease; (iv) predict effective treatment response [43,44]. The close contact with urothelium makes urine an attractive approach to detect the presence of tumor cells, in a minimally invasive way. Importantly, this liquid biopsy approach would allow multiple longitudinal sampling of the tumor to identify presence of malignancy, grade, and genomic landscape for improved clinical follow-up. [53,54].

**Figure 1:**
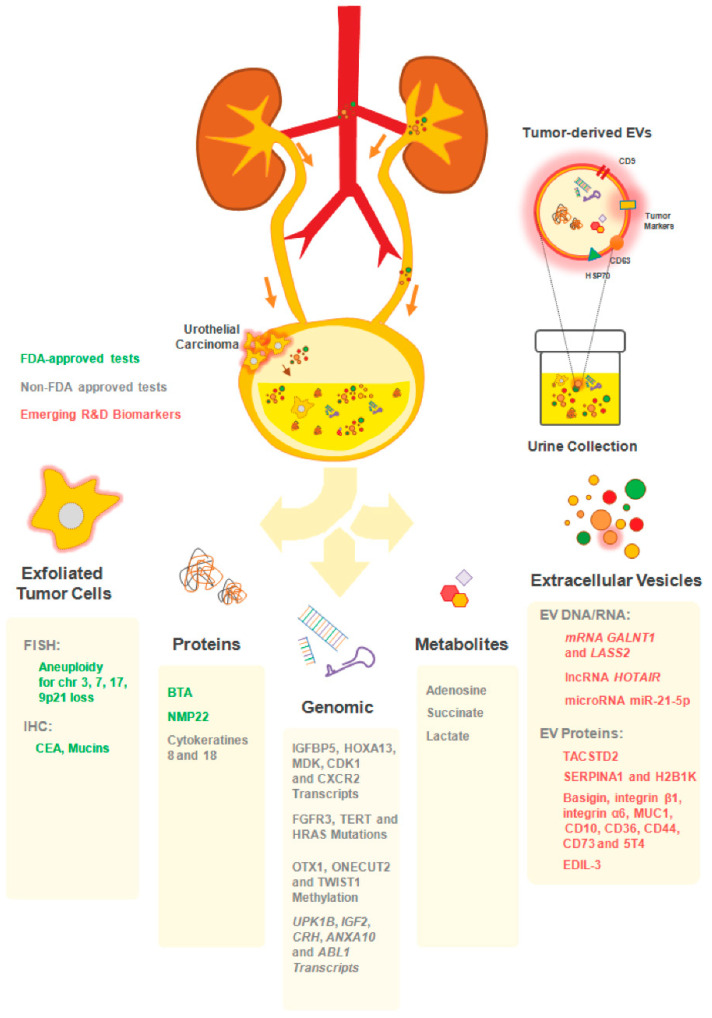
Urine biomarkers for bladder cancer (BCa) diagnosis and follow up. Illustration of the distinct available approaches for the detection of urothelial cancer cells in patients’ urine. The close interaction between the bladder tumor and the urine makes this body fluid a reliable source of cancer biomarkers. A plethora of non-invasive assays exploring distinct analytes (exfoliated tumor cells; proteins; genes; metabolites and extracellular vesicles) in patients’ urine allows the longitudinal analysis of tumor progression. Some of the commercially-available tests includes FDA-approved (UroVysion™: aneuploidy of chromosomes 3; 7; 17 and the loss of 9p21 by FISH; ImmunoCyt^™^/uCyt+^™^ test: detection of carcinoembrionary antigen (CEA) and mucins by immunohistochemistry (IHC); bladder tumor antigen (BTA) TRAK/BTA Stat and NMP22 BC test kit); non-FDA approved (CxBladder™: IGFBP5, HOXA13, MDK, CDK1 and CXCR2 by RT-qPCR; Assure MDx™: FGFR3, TERT and HRAS (mutations), OTX1, ONECUT2 and TWIST1 (methylation); XPert Bladder Cancer Monitor™: UPK1B, IGF2, CRH, ANXA10 and ABL1 by RT-qPCR; and UBC™: cytokeratins 8 and 18 by ELISA) and the emerging extracellular vesicles (EV)-based biomarkers (not commercialized yet).

Following this line of thought, previous research for BCa biomarkers has been conducted using mostly proteins, nucleic acids, inflammatory and metabolite markers, within the concept of liquid biopsies [55,56]. Taking into consideration that such biopsies concern the detection of any kind of molecular or cellular biomarkers in patient bodily fluids (including urine, blood, saliva, pleural, peritoneal, or cerebrospinal fluids), a novel biomarkers array emerged. These include circulating tumor cells (CTCs), proteins, metabolites, circulating nucleic acids, namely cell-free tumor DNA (ctDNA), messenger RNA (mRNA), micro RNA (miRNA), or long non-coding RNA (lncRNA). Most of these biomarkers may be found free or within extracellular vesicles (EVs) shed by tumor cells or by other elements of the tumor microenvironment [56,57] (Figure 1). There is a growing interest on the liquid biopsy concept, since (i) the biomarkers found have extensive potential for diagnosis and monitoring of disease stage and recurrence; (ii) prediction of therapeutic response/resistance and disease prognosis, with minimally invasive procedures, and (iii) helping therapeutic clinical reasoning based on identified molecular changes [56,57,58].

### 2.1. Commercially Available Urine Biomarkers in Bladder Cancer

Several interesting and promising biomarkers have been under clinical scrutiny during the past years, although only those approved by in vitro diagnostics (IVD) regulatory entities (e.g., FDA) became commercially available biomarkers, to be used as adjuncts to cystoscopy in primary diagnosis and follow-up of BCa. Taking into consideration the various reports on the subject, novel urinary biomarkers contributed to higher sensitivity but lower specificity than cytology, leaving them out of international guidelines recommendations [1,36,53,54,59,60,61,62]. Table 1 provides an overview of the biomarkers approved for clinical use and their reported diagnostic accuracy.

#### 2.1.1. FDA-Approved Urine Biomarkers

The bladder tumor antigen (BTA) is a complement factor H related protein secreted by malignant cells, which confers them survival advantage, as it interferes in the complement cascade [79]. There are two approved versions of this test for BCa follow-up in concurrent use with cystoscopy, the BTA TRAK and the BTA Stat (Polymedco Inc., Cortlandt Manor, New York, USA) [63]. In different reviews and meta-analysis, the BTA Stat has a sensitivity and specificity of 64% and 77%, respectively, whereas the BTA Trak has 65% and 74%, respectively [64,65,66]. The sensitivity was higher in the diagnosis of symptomatic patients rather than in follow-up, but with similar specificity. Both tests demonstrated higher sensitivity than urinary cytology, despite the decreased specificity in conditions where the complement factor H related protein is present, such as in other genitourinary malignancies and benign conditions with hematuria, including lithiasis, inflammation, instrumentation, and intra-vesical therapies [31,64,65,66,80].

The nuclear matrix has an important role on DNA replication and RNA transcription and splicing [81], with nuclear matrix proteins (NMP) being essential components of mitosis, with a role in tumoral proliferation. Numerous NMPs have been described in solid tumors, although NMP22 was shown to be specific for BCa [81,82]. It is released from apoptotic cells towards urine, with significantly higher release rate in cancer than in normal cells [81,83,84]. The NMP22 BC test kit (Matritech Inc.; Newton, MA, USA) is a quantitative test used for patient follow-up, whereas the NMP22 BladderChek test^®^ (Matritech Inc.; Newton, MA, USA) is qualitative and used for both follow-up and initial diagnosis, in symptomatic patients [85,86,87]. Concerning sensitivity and specificity, the quantitative test has 69% and 77%, while the qualitative has 58% and 88%, respectively [64,67,68,69,88,89]. When compared to urinary cytology, the sensitivity of NMP22 was higher (70% versus 40%), albeit specificity was lower (81% versus 97%) [28]. Taken together, both NMP22 and cytology, resulted in sensitivity of 91% [28,89]. Notably, Grossman et al. studied approximately 2000 patients, to compare NMP22 Bladder Chek test^®^ with cystoscopy, and observed decreased NMP22 sensitivity (50–56%) in comparison to cystoscopy (89–91%), although diagnostic accuracy was 94–99% if both tests were considered together [90]. Although NMP22 has higher sensitivity than urinary cytology, specificity is too low to replace it. The fact that it is released from apoptotic cells, which might also be seen in benign conditions, is responsible for the relatively high false positive rate [91]. However, if combined with cistoscopy, this significantly increases its diagnostic value.

The ImmunoCyt^™^/uCyt+^™^ test (Diagnocure Inc, Quebec, Canada) combines cytology with monoclonal antibody immunofluorescence labelling to detect three BCa antigens, M344, LDQ10, and 19A11, specifically found in malignant exfoliated urothelial cells [92]. To be positive, it requires many exfoliated cells (>500 per field). This test is expensive, with inter-observer variation and time-consuming analysis, but less prone to be influenced by benign inflammatory conditions, comparatively to other tests [93,94]. It is recommended in BCa patients only for follow-up as adjunct test to urinary cytology [95]. Sensitivity varies between 83% and 85% and specificity between 75% and 87%. These are higher in primary diagnosis than follow-up [28,64,65,70]. Mowatt et al. [28] compared uCyt+^™^ with urinary cytology and showed that this test presented higher sensitivity (82% versus 44%) and lower specificity (85% versus 94%), respectively. Interestingly, the simultaneous use of both tests improved sensitivity without impacting on specificity (87% and 68%, respectively). Sensitivity and specificity, in the study of Schmitz-Dräger et al. [96], was 85% and 88% for immunocytology and 84% and 98% for cystoscopy. When combined, sensitivity increased to 100%, whereas specificity decreased to 87%. Although less prone to interference, immunoCyt^™^ has lower specificity than urinary cytology. Likewise, despite combination with cystoscopy increases sensitivity, the false positive rate remains elevated [96]. Pfister et al. [97] assert that due to its good sensitivity, the combined use of uCyt+^™^ with cytology might delay the time intervals between cystoscopies, particularly in lower risk patients. Currently, this test was approved only for patient follow-up [95].

UroVysion (Abbott Laboratories, Abbott Park, Illinois, USA) is a fluorescence in situ hybridization (FISH) probe set to detect bladder cancer cells [95,98]. It uses fluorescent labelled DNA probes to assess genetic changes in exfoliated cells, namely chromosomal aberrations suggestive of BCa, aneuploidy of chromosomes 3, 7, and 17, and loss of the 9p21 locus. It has been approved for primary diagnosis and follow-up of BCa patients [95,98]. The reported sensitivity is 63–72% and specificity 85–87% [28,64]. Their diagnostic accuracy was superior in primary diagnosis than in follow-up, showing low sensitivity, similarly to urinary cytology, particularly for low grade tumors [71,72]. Compared to cytology, UroVysion had better sensitivity (72% vs. 42%) and lower specificity (83% vs. 96%) [71]. When used simultaneously, there was a significant improvement in sensitivity but still a low specificity of 50% [72,73]. UroVysion™ is more expensive than cytology and requires specialized laboratory techniques. However, it could be useful in situations of atypical cytology and equivocal cystoscopy, identifying patients that may need further investigation [62,97]. Two prospective studies found that UroVysion had high positive predictive value, supporting that patients with a positive test and negative cystoscopy are more likely to have disease recurrence within one year [99,100,101]. Thus, a FISH test that is positive may be used to anticipate BCa recurrence during follow-up, especially in low risk patients [99,100], and reduce the number of unnecessary bladder biopsies [102]. Therefore, these studies suggest that chromosomic aberrations precede the detection of malignant lesions by cystoscopy and other standard techniques [101].

Analyses comparing the above-mentioned biomarkers have been reported. No differences were found in terms of sensitivity and specificity between the NMP22 test kit (cut-off > 10 U/mL) and the BTA Stat, in different stages and tumor grades [91,103,104,105,106,107,108]. The ImmunoCyt^™^ has higher sensitivity for low stage (Ta, T1) and low-grade tumors, although lower specificity than the UroVysion^™^ test [107,109,110]. However, although these tests were FDA approved for diagnosis and follow-up of BCa, together with standard techniques, most of these studies are case–control ones in populations with high prevalence of the disease, giving them an unrealistically high positive predictive value. On the other hand, the question remains how to interpret positive findings of these tests, when no significant findings are found on cystoscopy during follow-up. In fact, most positive results have not been submitted to confirmatory biopsy. Moreover, there are few external validation studies to support their use in daily practice. In summary, multicentric prospective studies are required to assess consequences from positive and negative tests in the long term, to increase the likelihood to be supported by international urology organizations. 

#### 2.1.2. Non-FDA Approved Urine Biomarkers

To overcome the limitations of approved diagnostics biomarkers, extensive research is ongoing to find more effective biomarkers for BCa diagnosis and follow-up. There are several commercially available tests, despite not being approved by regulatory institutions. The CxBladder (Pacific Edge Diagnostics, Dunedin, New Zeland) is a RTqPCR test in voided urine, that quantifies different mRNAs expressed in BCa, as *IGFBP5*, *HOXA13*, *MDK*, *CDK1,* and *CXCR2*, associated with non-malignant conditions, to reduce the number of false-positives results due to inflammation [75,111]. The Triage^™^, Detect^™^, and Monitor^™^ tests have specific population targets. The first was developed for screening of high-risk populations as a pre-test guiding the need for cystoscopy, the Cxbladder Detect^™^ was intended for aiding in diagnosis of symptomatic patients and the Monitor^™^ for BCa follow-up [75]. Studies using Cxbladder Detect^™^ found higher sensitivity but lower specificity than cytology (73.6% sensitivity and 81.7% specificity) in one study [74], while another described 82% sensitivity and similar specificity [112]. There are reports for Cxbladder Monitor™ stating a sensitivity of 93% that increases to 95% in high risk patients [75]. A large study comparing biomarkers performance for BCa detection in urine, found that the Cxbladder Monitor^™^ sensitivity (91%) overcomed cytology by 22%, NMP22 BC test kit^®^ by 26% and NMP22 BladderChek^®^ by 11%, with an estimated reduction in the number of cystoscopies needed in follow-up by 81.7% [38]. Although prospective confirmatory trials are needed, some authors suggest its use as an auxiliary test to postpone the need of repeated cystoscopies in low risk patients [38,54]. The Assure MDx^™^ (MDx Health, Irvine, CA, USA) is a test performed in urine to identify DNA mutations in three genes (*FGFR3*, *TERT,* and *HRAS*) and methylations in another three genes (*OTX1*, *ONECUT2,* and *TWIST1*) [113]. A multicentric study demonstrated a sensitivity of 93% and specificity of 86% for BCa diagnosis [76]. It might be useful for screening low risk patients with symptomatic hematuria, reducing by an estimated 77% the number of unnecessary diagnostic cystoscopies. The XPert^®^ Bladder Cancer (BC) Monitor (Cepheid, Sunnyvale, CA, USA) is a RT-PCR test that measures the number of urinary transcripts in five genes, *UPK1B*, *IGF2*, *CRH*, *ANXA10,* and *ABL1*, and was designed for BCa patient follow-up [77]. This test was superior to cytology on NMIBC during follow-up, in terms of sensitivity (84% versus 33%), while presenting similar specificity (91% versus 94%) [77], despite controversial findings from another study, that indicated 46.7% sensitivity and 77% specificity [114]. The heterogeneity between studies and the lack of external validation makes its present use unreliable. The UBC^®^ (Urinary bladder cancer) is a test that detects the expression of cytokeratins 8 and 18 in urine, with presentation of quantitative, UBC^®^-ELISA, and qualitative UBC^®^-rapid procedures [78]. The reported sensitivity for UBC^®^-rapid was 86.9% for detecting Cis, 30.4% for low grade NMIBC, 71.4% for high grade NMBC, and 60% for MIBC [78]. Other studies reported sensitivities between 30% and 87% for Cis and specificity of 63–91% [115,116,117,118]. The UBC^®^-rapid is a test that provides results within 10 min, but in comparison with other tests has the lowest specificity [115]. 

### 2.2. Emerging Urine Biomarkers

Besides these commercially available diagnostic tools for BCa detection in the urine, extensive research is underway to find more effective biomarkers [60,86,119]. The insufficient number of patients in most studies, the lack of external validation in large scale prospective studies, and absence of comparative trials between biomarkers, foster the need for both methodological improvement of existing biomarkers and uncover novel robust biomarkers. Moreover, the existing biomarkers, in general, perform poorly in low risk BCa or have low specificity, and are more accurate in the initial diagnosis of BCa than in follow-up [66]. Taken together, these limitations preclude actual recommendations by most international clinical societies, and current literature suggests that single biomarkers are insufficient to overcome this problem. Therefore, the current trends of research are focusing on the combination of different biomarker signatures, to develop more accurate diagnostic and surveillance tools in BCa, as well as to predict its behavior in order to provide prognostic information [120].

## 3. Extracellular Vesicles from Liquid Biopsies as a Source of Biomarkers

Recently, tumor-derived extracellular vesicles (EVs) have received considerable interest by the biomarker research community for BCa diagnosis and follow-up. EVs are non-replicable small lipid bilayer membrane vesicles continuously released by all prokaryotic and eukaryotic cells to the extracellular surroundings. Importantly, this mechanism allows cells to exchange information (encoded in nucleic acids or proteins) between donor and target cells [121].

**Figure 2:**
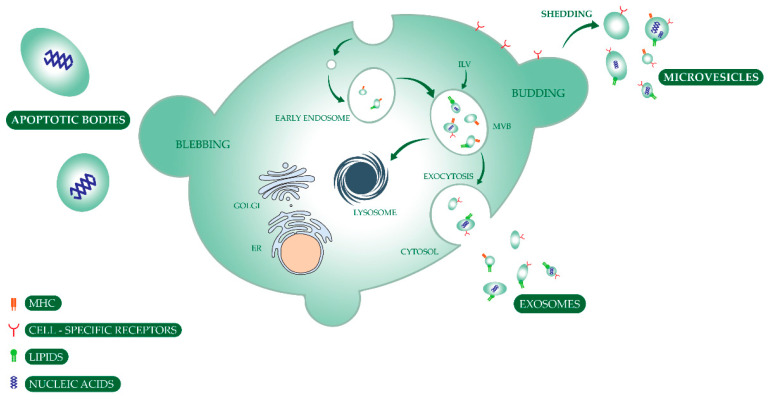
Schematic representation of extracellular vesicles biogenesis. Extracellular and plasma membrane molecules are engulfed by plasma membrane endocytosis, creating the early endosomes. These are converted into late endosomes called multivesicular bodies (MVB) containing intraluminal vesicles (ILV). The MVBs may either fuse with the plasma membrane and empty their ILVs by exocytosis, termed exosomes, or may be converted into lysosomes and degrade their components. The process of microvesicle formation is calcium dependent and comes from direct shedding from outward cellular membrane budding; thereby carrying membrane markers of the parent cell. Apoptotic bodies are produced by secreting cells undergoing programmed cell death. Extracellular vesicle uptake by recipient cells may occur via fusion of the vesicle membrane with the cell membrane or by endocytosis. The vesicle may also transduce an intracellular signal by ligand binding to a receptor on the recipient cell. Abbreviations: MHC—major hystocompatibility complex; ER—endoplasmic reticulum; MVB—multivesicular bodies; ILV—intraluminal vesicles.

Depending on their biogenesis mechanism and secretion, EVs are broadly divided in exosomes, microvesicles, and apoptotic bodies (Figure 2). Exosomes are the generally smallest vesicles (30–150 nm) and originate by inward budding of intracellular endosomes, later converted into multivesicular bodies (MVBs) that fuse with the cellular membrane and release their cargo into the extracellular space. Instead, microvesicles are typically larger (100–1000 nm) and shed directly from the bleebing of the outward cellular membrane [46,121,122,123]. Apoptotic bodies (1000–5000 nm) are produced by cells undergoing programmed cell death. The processes of synthesis and release of EVs are regulated by endosomal sorting complexes required for transport (ESCRT), p53/TSAP6 pathway, syndecan-syntenin-ALIX, Rab proteins, phospholipase D, sphingomyelinase, and ceramide [124]. To date, it is not possible to experimentally support the attribution of certain activities or markers to specific EV subtypes, which prompted the International Society for Extracellular Vesicles (ISEV: www.isev.org/) to publish guidelines on EVs nomenclature and characterization. The current recommendation is to report all EV subtypes generically as “extracellular vesicles”, while describing in detail the mechanisms used for their separation and characterization, their physical characteristics, biochemical composition, or descriptions of the cell of origin, unless their biogenesis pathway is confirmed [124,125,126].

### 3.1. Physiological Functions of Extracellular Vesicles 

EVs behave ubiquitously, and have been identified in most body fluids, including blood, saliva, breast milk, urine, and amniotic fluid [125,126,127,128,129,130,131,132]. It is known that they carry a cargo from their donor cell, primarily composed of proteins, mRNAs, miRNAs, lncRNAs, small DNA fragments, and lipids. Indeed, it was postulated that EVs may reflect the biological functions of the originating cells [46,123,133], even though they were thought to be biologically insignificant or a simple vehicle for cellular waste disposal. In recent years, EVs were recognized as having physiological and pathological relevance [134]. 

**Figure 3:**
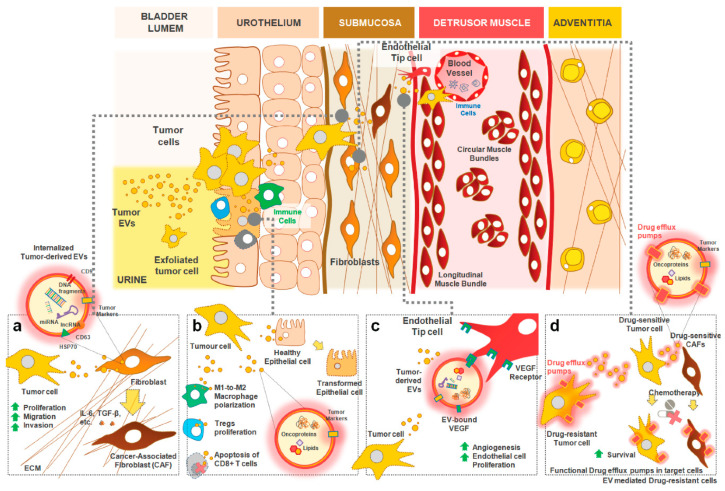
Routes of EV delivery to target cells and their potential role in bladder cancer progression. (**a**) Transfer of EV-enclosed nucleic acids derived urothelial carcinoma cells to nearby naïve fibroblasts can induce their transformation into Cancer-Associated Fibroblasts (CAFs) with altered pro-tumoral secretome. (**b**) Likewise, transfer of EV-associated lipids and/or oncoproteins to neighbor or distant cells modulate targeted immune cells into an immunosuppressive phenotype and/or facilitate the transformation of healthy epithelial cells. (**c**) Many of the signaling molecules that integrate the EVs membrane can act directly on the surface receptors of target cells and trigger their own cell signaling pathways without the need of EV internalization. (**d**) The uptake of EVs derived from drug resistant tumor cells by drug sensitive tumor cells (or even supporting stroma) can mediate the transfer of functional receptors/molecules to recipient cells, thus allowing a similar phenotypic behavior in cells that originally lacked those receptors/molecules (e.g., transfer of functional drug efflux pumps).

Extracellular vesicles are key intervenients in several processes involved in cellular homeostasis [134]. Besides their physiological role on cell survival and anti-stress protection, they have a main function in intercellular communication, transporting key molecular messengers to recipient cells, thereby influencing the recipient cells function. Packaging this information into vesicles provides additional protection to the messengers (i.e., cargo) and allows simultaneous delivery to remote locations, which might be achieved through distinct mechanisms (Figure 3): (i) transfer of nucleic acids that induce phenotypic changes and affect multiple functions in recipient cells; (ii) transfer of lipids and proteins (such as cytokines, chemokines, and growth factors) to neighboring or distant cells, thus modulating the targeted recipient cells; (iii) trigger cell signaling pathways in recipient cells by exposure of ligands, proteins, and lipids, that bind to and stimulate matched receptors at the cell surface; (iv) transfer of functional receptors to recipient cells, thus allowing cell signaling in recipient cells that originally lacked that receptor or enhancing their number [47,48,49].

Extracellular vesicles have been involved in thrombosis and hemostasis, as they contribute towards increased availability of negatively charged phosphatidylserine and tissue factor, thus providing the triggers for activation of the extrinsic pathway and subsequent thrombin generation, promoting pro-thrombotic effects and platelet aggregation [48]. Interestingly, bladder cancer patients are at increased risk for venous thromboembolism [135], an event associated with the worst prognosis. Similarly, the crosstalk between blood and endothelial cells mediated by EVs expose cell surface receptors and adhesion molecules, required for angiogenesis and neovascularization, of relevance upon tissue injury, post-ischemic revascularization, and regeneration [47,48,49]. A pleiotrophic effect of EVs on immunoinflammatory processes has also been described. Dendritic cell-derived EVs enhance natural killer cell cytotoxic activity and stimulate epithelial cells to release proinflammatory cytokines [49]. Others, derived from circulating leukocytes, participate in endothelium activation and upregulate the release of adhesion molecules, leading to leukocyte recruitment; EVs may also have antigen-presenting properties, exposing major histocompatibility complexes (MHCs) and co-stimulatory molecules on cell surface, initiating a pro-inflammatory response in epithelial cells and T-cell activation. Dendritic cell-derived microvesicles containing tumor necrosis factor-α (TNF-α) might initiate an innate immune response in epithelial cells and affect adaptive immunity, whereas platelet-derived microvesicles can increase B-cell production of immunoglobulins and activation of the complement system [47,48,49].

### 3.2. Pathological Functions of Extracellular Vesicles in Bladder Cancer

Interestingly, the recognized function of EVs as critical effectors in the maintenance of physiological cell-to-cell interactions seems to be severely disturbed throughout cancer progression. Indeed, the transfer of pro-tumoral EVs cargo between cancer cells and the surrounding tumor microenvironment influences the multiple stages of tumorigenesis, namely neoplastic transformation, proliferation, migration, invasion, and metastasis to distant organs, angiogenesis, immune response, and emergence of drug resistant traits in cancer cells [47,121,136] (Figure 3). Their potential for tumor initiation was shown in prostate cancer, where EVs isolated from tumor cells were able to upregulate the pro-survival protein STAT3, converting normal into malignant epithelial cells [137]. Similarly, in other cancer models, *BRCA1*-KO fibroblasts treated with sera (containing EVs) from cancer patients yielded higher proliferation and malignant transformation than wild type control fibroblasts [138]. This was demonstrated in BCa as well, as healthy recipient fibroblasts gained malignant phenotypes and transformed into cancer associated fibroblasts (SMA, FAP, galectin), after exposure to cancer cell-derived EVs [139]. EVs were also able to promote tumor cell proliferation in several cancers, including BCa. When derived from cells under hypoxic conditions, EVs carry high levels of lncRNA-urothelial cancer associated 1 (lncRNA-UCA1), which promotes proliferation and invasion in human recipient cells [140]. Their ability to induce migration, invasion, and angiogenesis has also been demonstrated [141]. In fact, tumor-derived EVs can promote angiogenesis by supporting communication between cancer and endothelial cells. Indeed, extracellular vesicles isolated from high grade bladder cancer cells, and from the urine of patients with high grade bladder cancer, contained EDIL-3 and increased angiogenesis and migration of bladder cancer and endothelial cells. Indeed, when EVs originate from high grade bladder cancer cells isolated from the urine of patients submitted to radical cystectomy, EVs contain substantially higher levels of EDIL-3, a pro-angiogenesis and migration protein, than healthy controls. Following EDIL-3 knock-down in bladder cancer cells, the collected EVs had lower EDIL-3 levels, and were unable to promote angiogenesis and migration [141]. Likewise, the EVs isolated from MIBC cells had increased levels of periostin, which was capable of activating ERK oncogenic signals. This resulted in increased aggressiveness of low-grade tumor cells and was associated with worse prognosis, in both MIBC cell lines and clinical tissue samples [142]. On the other hand, Franzen et al. [143] demonstrated the ability of MIBC derived EVs to induce epithelial to mesenchymal transition, a well-known mechanism for initiation of metastasis and cancer progression. In another study, Ostenfeld et al. reported that altered secretion of EVs containing tumor suppressive miRNAs, like the miR-23b, regulated by members of the RAB family, namely the RAB27A and RAB27B, contributed to invasion, anoikis, angiogenesis, and pulmonary metastasis in BCa patients [144]. The increased amount of mucin-1 (MUC1) and epidermal growth factor (EGF) receptor HER3 in EVs were shown to be associated with a more favorable prognosis [145]. The EVs have a prominent role in damping the hosts immune cell response to the emerging cancer cells [146]. Indeed, their role in immunosuppression has been well established in several cancer types [146], once they may cooperate with cancer cells to overcome immune checkpoints [146]. Nevertheless, the specific contribution of EVs for immunosuppression of host response in BCa remains poorly understood. 

### 3.3. EV Separation and Characterization from Urine Samples: Pitfalls and Technical Considerations

#### 3.3.1. Pre-Analytical Considerations

Currently, there is limited consensus on the standard pre-analytical procedures for the clinical validation of EV-based diagnostics in bladder cancer [147,148]. Some of these pre-analytical factors include the standardization of procedures for urine selection, collection, storage, and shipping/transportation conditions. Noteworthy, many of these factors have a direct impact on the co-elution and polymerization of Tamm–Horsfall protein (THP), one of the major contaminants of urinary EV separation. Indeed, the (i) timing of urine collection (e.g., first morning urine vs spontaneous urine vs intravesical urine); (ii) the need for inter-day urine collection due to sample variation; (iii) the necessity for stabilization of urine pH; (iv) the type and impact of THP inhibitor cocktails added to the urine (e.g., reducing dithiothreitol (DTT), detergent CHAPS or urea); (v) its storage temperature (e.g., 4 °C vs 20 °C); and (vi) maximum storage time prior EV separation are some of the unresolved issues required to improve EV-based diagnostic accuracy. 

So far, the consensus seems to prefer first morning urine for collection, with some reports stating that a citrate-based buffer is beneficial for controlling urine pH while reducing THP protein precipitates after thawing [148]. Nevertheless, such pre-analytical interventions may affect the size and even the native composition of isolated EVs [131,149,150]. Additionally, the high variation of EV recovery due to inter-day urine collection seems to be hampered by the addition of protease inhibitors [148]. Unfortunately, the optimal inhibitor cocktail is still an open debate with several authors showing that different protease inhibitor cocktails had different impacts on the ratio of THP polymerization and consequently on the yield of urinary EVs in the UC pellets [148,151,152,153]. 

Regarding urine storage/transportation, 4 h seems to be the maximal time interval upon urine collection to minimize EV sample degradation. Importantly, this time interval seems to be heavily reliant on the storage temperature and type of analyte (e.g., nucleic acids, proteins, lipds, etc.) [154]. 

Taken together, several studies show that several pre-separation methodological issues have a remarkable influence on the yield, purity, and cargo profile of EVs isolated from urine samples [148,154,155]. Importantly, this impact is transversal to all studies regardless of the selected EV separation method. Indeed, the standardization of pre-analytical variables is required to ensure a reliable evaluation on the reported quantity and EV profile for a given pathological state. Moreover, the development of shared Standard Operating Procedures (SOPs) would enable the comparison (and even merging the data) of urine EVs derived from bladder cancer patients from different laboratories. This would facilitate the establishment of multicentric clinical trials to validate the clinical feasibility of EV-based biomarkers. Considering this, the International Society of Extracellular Vesicles (ISEV) has been supporting several initiatives to favor the development of such SOPs [156]. Some of these initiatives includes the Minimal Information for reporting EV-related research [124], the EV TRACK: EV Transparent Reporting and Centralizing Knowledge [157], and the Clinical Wrap-Up session at ISEV2018 [158]. 

#### 3.3.2. EV Separation Methods 

Regarding EV separation from urine samples, several technologies can be used for this purpose. Some of the conventional methodologies include differential ultracentrifugation (dUC) [159], density gradient ultracentrifugation (gUC) [160], chemical precipitation [161], affinity capture [162], hydrostatic filtration dialysis [163,164], ultrafiltration (UF) [155,165,166], and size exclusion chromatography (SEC) [167]. With no perfect solution in sight, the selection of the optimal EV separation method typically lies on the compromise between urine EV recovery yields, EV integrity, and purity [124,168]. Within this reality, dUC remains the most used methodology despite having a low recovery yield of EVs (1–5%) from urine samples. Moreover, dUC has a reported co-precipitation of contaminants (e.g., urine proteins, cell membrane debris, etc.) with the pellet EVs. This issue may compromise a reliable proteomic approach for biomarker discovery [160]. 

Even more, gUC have a reported EV recovery yield of nearly 30% from crude urine samples. However, both ultracentrifugation-based approaches have a timely and laborious nature which may compromise its generalization for some clinical applications.

Facing this bottleneck issue, several authors have pursued other approaches for isolating highly pure EVs from urine samples. In this regard, several two-step combination methods (e.g., UF combined with SEC or asymmetrical-flow field-flow fractionation) have been pursued to minimize the impact of urine contaminants on the purity of isolated EV sample [165,169,170,171]. Unfortunately, despite having higher EV recovery yields (up to 60%) these techniques fail to provide the same reliable EV proteomic analysis obtained via gUC gold standard [165,171]. 

Direct comparisons between the different pre-analytical factors and EV separation methods on the observed results remain difficult to perform. This is mostly due to inter-laboratory variability, lack of widely accepted EV-marker normalization methodologies, backed up by the limited biological sample availability to test all these conditions in a single experiment. Recently, the use of “spike-in” fluorescent EVs standards were proposed to monitor the efficacy of pre-analytical methods and EV separation procedures [172]. Their use could enable data normalization across laboratories and a deeper comprehension on the causes of variability in the urinary EV cargo. This would increase the detection rate of artefacts originated by technical variation (e.g., sample preparation and instrumentation). Indeed, the use of such internal EV standards would facilitate the establishment of consensual and evidence-based SOPs for optimal collection, storage, and handling of urine EVs [148]. 

As far as new urine EV separation methods are concerned, microfluidic [173,174,175,176] and/or nanofiltration [177] miniaturized systems have recently emerged as promising approaches. These technologies separate efficiently EVs from other urine components based either on acoustic trapping [174], lateral fluidics displacement [175], immunocapture [178] and/or physical entrapment by nanowires technology [176] or double filtration meshes [173,177]. These new technologies have the advantage of enabling easy and rapid sample processing (e.g., less than 30 min) with minute amounts of urine sample (up to 1 mL), compatible with an -omics approach. These are some of the critical features of future point-of-care devices intended for clinical use. 

#### 3.3.3. Single-EV Detection Technologies 

The EV-based liquid biopsy concept for bladder cancer diagnosis relies on the detection of rare EV-subsets (shed by bladder cancer cells) in the pool of isolated urine EVs (derived from virtually all cells of the body). In most cases, only small amounts of clinical samples are available, rare molecular targets have to be detected in complex biological fluids with high specificity and sensitivity in a timely fashion. Indeed, most of traditional methodologies (NTA, TEM, WB, etc.) for EV analysis become obsolete and fail to provide such diagnostic detail under these strict clinical requirements [156].

To fulfill such requirements, innovative optical [179,180,181], nano-flow cytometry [182,183,184,185], Raman [164,186,187] and other plasmonic sensors methods [188,189,190,191] have recently emerged for highly sensitive single-EV detection. Nevertheless, their application has not been applied to urine samples and the analysis of single-EVs and other submicron particles has presented many challenges and has produced a few controversial results in other types of samples. Thus, consortium-based efforts are being currently implemented. This will allow a combined effort for technique optimization for EV detection, definition of data reporting criteria, and finally to forge consensual international guidelines for each technique prior clinical application [192].

### 3.4. EVs as a Source of Biomarkers for Bladder Cancer Diagnosis

The role of EVs in the maintenance of tissue homeostasis, and the demonstration of their disruptive role during cancer progression and metastization, render them as an attractive source for diagnostic biomarker research, particularly in bladder cancer. Indeed, EVs have several advantages as source of cancer biomarkers. Firstly, some studies suggest that EVs secretion by tumor cells may be higher than by non-tumor cells, even though this still needs to be proved since other studies failed to demonstrate such association [193]. Secondly, the EVs presence and stability in large quantities in most human body fluids, being more abundant in liquid biopsies than CTCs, makes them easily attainable for non-invasive collection and their detection is technically feasible [45,194,195]. Thirdly, their cargo reflects the biological behavior and composition of their donor cells and, thereby, they may carry molecular signatures associated with specific phenotypes or therapeutic resistance patterns [45,46,194,195]. Finally, the lipid bilayer membrane protects their cargo against degradation [45,130,195]. EVs recently emerged as a source of biomarkers in cancer diagnosis and management [45,195,196,197,198,199,200]. EVs isolated from urine and blood have shown specific miRNA, mRNA, and protein content in different types of solid tumors [201,202,203,204,205,206]. Notably, research and knowledge on EVs is now expanding to other diseases such as hepatitis C, chronic kidney disease, and central nervous system and cardiac diseases [207,208,209,210].

Using EVs as diagnostic tool in bladder cancer remains elusive, however, a large body of evidence is now accumulating and demonstrating their potential as a source of biomarkers for non-invasive diagnosis of BCa. Research has been conducted on protein and genetic content of EVs from patients with bladder cancer, providing a library for future biomarker identification [196,197,198,199,200] (Table 2). Although plasma or serum can be used, urine is the preferred body fluid for EV collection, due to its availability, low invasive procedure, and its physical contact with the bladder tumor cells. Recently, a high number of EVs were detected in the urine of BCa patients when compared to healthy controls, using a newly developed double-filtration microfluidic system as a point-of-care diagnostic device, which displayed a sensitivity of 81% and a specificity of 90% [174]. 

The characterization of genomics in urinary EVs, contributed towards the growing interest in its RNA content. Indeed, miRNA and lncRNA are small non-coding RNAs that regulate the expression of protein-coding genes involved in several cellular processes, including tumor development and progression [211]. Their presence in urine and other body fluids, either in their cell free form or as part of EVs, makes them interesting sources of tumor marker research with diagnostic, treatment, and prognostic objectives in bladder BCa [212]. For this purpose, Perez et al. [213] compared the urinary EV transcriptome from BCa patients and healthy controls, by performing PCR analyses of 15 genes with differential expression between both groups. The authors described four genes differently expressed in urinary EVs, where *GALNT1* and *LASS2* were specific of cancer patients, and the *ARHGEF39* and *FOXO3* transcripts were detected only in healthy controls. Other studies analyzed lncRNAs isolated from EVs, finding different genetic patterns in patients with MIBC in comparison to normal controls, particularly the HOX transcript antisense RNA (*HOTAIR*), implicated in tumor initiation and progression [214,215]. The interest in this panel of genetic biomarkers was later confirmed in another study that showed association to disease recurrence and poor prognosis [216]. Others studied the miRNA content of urinary EVs from BCa patients [217,218,219,220,221] and found miRNA signatures characteristic of high-grade BCa [216,220], which can be suggested as biomarkers of advanced disease. Interestingly, a study detailed one miRNA (miR-21-5p) overexpressed in urinary EVs of BCa patients with 75.0% sensitivity and 95.8% specificity for detecting disease, which was still present despite negative urinary cytology, suggesting it might detect BCa at an earlier stage of the disease, with no cytological changes [220]. 

Although most of the genomic research includes RNA, EVs may also be a source of tumor DNA. Lee et al. compared the genomic profiling of ctDNA and EVs DNA with tumor samples of nine patients submitted to radical cystectomy, and found the amplification of *MDM2*, *ERBB2*, *CCND1,* and *CCNE1*, and deletion of *CDKN2A, PTEN,* and *RB1* genes, thus suggesting EVs DNA could also be another source for liquid biopsy [222].

Proteomic analysis of urinary EVs content has also started to contribute with the identification of proteins, regarding its diagnostic properties. One of the first studies documented the protein content of urinary EVs from healthy donors, using liquid chromatography-tandem mass spectrometry [131]. Chen et al. [223] analyzed the EV protein content in the urine of patients with BCa, compared with inguinal hernia patients used as controls. Liquid chromatography-tandem mass spectrometry identified 107 differently expressed proteins, including tumor associated calcium-signal transducer 2 (TACSTD2), a cell-surface protein absent in blood and with minimal expression in normal cells. Another study analyzed proteomic data from 129 BCa patients and 62 healthy controls and revealed urinary BCa biomarkers for diagnosis (alpha-1 antitrypsin, SERPINA1) and prognosis (Histone H2B type 1-K, H2B1K) [224]. Smalley et al. [225] using mass spectrometry found higher urinary levels for eight proteins in EVs from BCa patients. Besides the alpha subunit of GsGTP binding protein, resistin, and retinoic acid-induced protein 3, these authors identified five proteins associated with the epidermal growth factor receptor (EGFR) pathway, namely mucin 4, the epidermal growth factor receptor kinase substrate 8-like protein 1 (EPS8L1), the Eps15 Homology (EH)-domain-containing protein 4, the epidermal growth factor receptor kinase substrate 8-like protein 2 (EPS8L2) and the Guanosine-50-triphosphate hydrolyzing enzyme NRas (GTPase NRas). Another study, by Welton et al. [196] analyzed the urinary protein content on EVs of HT1376 bladder cancer cells, as well as in patients diagnosed with bladder cancer and healthy controls, and found several proteins elevated in BCa patients, namely basigin, integrin β1, integrin α6, MUC1, CD10, CD36, CD44, CD73, and 5T4. As mentioned before, Beckam et al. [141] found BCa derived EVs had higher levels of EDIL-3, which besides promoting angiogenesis and migration in a neoplastic environment, could also serve as a prognostic biomarker. The same principle could apply to periostin, which promotes tumor aggressiveness and progression, and is also present in higher levels in urothelial cancer patients. Its presence in cancer patients was associated with a poorer clinical outcome [142].

Further research on EVs is warranted, while creating the bases for accumulating evidence from past and present studies. Results should be consistently deposited in public databases, to facilitate the progress of research in this area. There are different compendiums available, namely webdomains such as the ExoCarta (http://www.exocarta.org), EVpedia (http://evpedia.info), and Vesiclepedia (http://www.microvesicles.org) which are updated databases on EVs characterization, content of proteins, mRNA, and lipids [226,227].

## 4. Discussion and Future Perspectives

Diagnosis and follow-up of bladder cancer currently relies on cystoscopy and cytology, despite known limitations. Cystoscopy is costly, invasive, and has reduced sensitivity for Cis or non-papillary lesions, whereas cytology lacks sensitivity for low grade tumors. Many urine-based tests have been developed to improve efficacy beyond current diagnostic tests. FDA-approved systems for diagnosis and monitoring of BCa have demonstrated higher sensitivity but lower specificity than cytology, particularly in cases of low grade and early stage or recurrent BCa; other tests are costly, limiting their use in the daily health practice (e.g., UroVysion test). Besides FDA-approved tests, the other commercially available tests remain mostly at the research level. Most tests have been assessed in inadequate conventional case-control studies, emphasizing the need for prospective cohort studies, with serial samples at different time points from a person at-risk, as well as large randomized trials, validating the biomarker clinical benefit compared to actual gold standard methods. This is the reason why their use as adjunct or surrogate to conventional cystoscopy and cytology is still not recommended by international societies’ guidelines. Moreover, positivity or equivocal results in these tests, when associated with negative cystoscopic findings, may increase patient’s anxiety and trigger further invasive medical examinations, namely biopsies or ureteroscopic procedures. Consequently, besides the need of adequate studies to validate biomarkers for early detection, the current trends of research should focus on the combination of biomarkers into signatures.

Extracellular vesicles released by cancer cells carry potential cancer specific biomarkers, as they shed directly from tumor cells and contain protein and nucleic acid material that reflect their cells of origin. EVs play indeed a fundamental role in intercellular communication, being key effectors in normal and pathophysiological cancer progression. Notably, unlike biopsies of solid tumors that provide a small picture of tumor heterogeneity, EVs might provide a wider perspective of tumor heterogeneity, since they are shed by tumor cells and from the cells of the tumor microenvironment. Moreover, their stability within the biological milieu, seemingly increased concentration in some tumors, and unique molecular signatures in oncological patients, makes EVs attractive biomarkers for cancer diagnosis and follow-up. Although out of the scope of this review, EVs can also be explored as therapeutic adjuvants, as a conveyance means for drug delivery and chemosensitization in BCa.

Here, we sought to describe research that has been done using EVs as biomarkers in BCa. Indeed, EVs hold promise as biomarkers, not only in BCa, but overall, in oncology. Nevertheless, further research from bench-to-bedside is still needed, as discussed next, particularly in demonstrating its clinical effectiveness. 

The first limitations to overcome are technical problems related to EVs separation and characterization. There is a need for consensus in EVs nomenclature, to eventually stratify exosomes, microvesicles, and apoptotic bodies [228], and the need for fast, reproducible, and effective separation methods, improving standardization and comparison between studies. Currently, the most frequently used methods for separation of EVs rely on ultracentrifugation procedures that separate them based on size and density [150]. However, this is a time-consuming, laborious, and expensive method, that needs large amounts of sample material and requires expensive equipment, halting its applicability in the clinical laboratory. Other procedures, including double-filtration microfluidic chip-based devices to separate EVs concentration at the point-of-care [174,229] have been proposed, which combine immuno-affinity, sieving, and trapping to concentrate EVs. Indeed, this approach has the advantage of needing lower sample volumes but the disadvantages of EVs structural damage and lower recovery rates, thereby hampering its clinical applicability. Further separation techniques include immune-affinity capture, using antibodies directed against EVs surface markers and bypassing ultracentrifugation [230]. 

Another overlooked technical issue regards the specificity of these different EVs separation methods. Some of the isolated material identified as of EVs origin by these methods may not in fact be EVs related but derived from other soluble urinary components. On the other hand, urine is enriched in contaminants, such as albumin, Tamm–Horsfall protein and different lipoproteins, and other substances that need to be fully identified and isolated [160]. Therefore, there is the need of a specific, reliable, standardized, and reproducible method, to reduce the confounding effect these contaminants on the EV separation process. A recent research identified a list of 684 of these potential contaminants and developed a bottom-up density gradient centrifugation method to separate EVs from different kinds of protein material in urine, with high specificity and methodological repeatability [160].

Before clinical application, standardization of pre-analytical conditions for handling urine specimens is also required. Variables such as urine collection, use of protease inhibitors, storage, and shipping conditions should be accounted for, albeit often disregarded [155]. Bridging clinical usefulness with EVs research requires reporting guidelines to support readability, interpretation, and replication of experiments. We strongly encourage researchers to follow the International Society for Extracellular Vesicles (ISEV) guidelines [124] and the recently created EV-TRACK database (http://evtrack.org) stimulates researchers to report their methodologies for developing standardized protocols, place experimental guidelines into practice and increase research reproducibility [157,231].

From a clinical analytical perspective, the use of EVs as a source of biomarkers needs to be properly validated in negative controls, since urinary EVs are produced by cells from all urinary tract. Indeed, one of the main impairments of available urinary biomarkers is the low specificity. Positive results with these tests might also occur in benign conditions such as benign prostatic hyperplasia, urinary lithiasis, endourologic stents, or urinary tract infections. Therefore, to overcome these limitations it is necessary to distinguish EVs derived from BCa cells, from other sources of EVs such as the kidney or prostate, imposing the need for including in clinical studies negative controls, such as EVS from patients with prostate and kidney cancer and hematuria, in order to fully determine the specificity for BCa. 

Another source of criticism that hampers EVs application as a source of biomarkers in BCa relies on the fact that most studies typically have a small and often heterogeneous cohort of BCa patients, limiting validity and comparisons. However, this is also one of the main limitations of other commercially available urine tests, that preclude their recommendation by most international scientific and clinical organizations. After the identification of robust candidate biomarkers in EVs, additional external validation in large independent multi-institutional studies are required to establish their value as useful biomarkers in bladder cancer.

## 5. Conclusions

Cystoscopy and cytology remain the clinically approved diagnostic and follow-up procedures for bladder cancer management. This review provides a critical insight into the available urine-based biomarkers, revealing their low improvement on the precision of diagnosis due to low specificity and limiting clinical utility, and fostering the need for more reliable, sensible, and specific urinary biomarkers for BCa.

Extracellular vesicles secreted from their cells of origin are vital players in the physiological and pathological intercellular communication processes and are known to promote cancer progression. In recent years, there has been growing interest in EVs as a source of biomarkers in liquid biopsies for cancer diagnosis, management, prognosis, and even as vehicles for cancer treatment. Published reports yielded encouraging findings, even though the path from bench-to-bedside still needs to be optimized, namely regarding standardizing separation protocols and including powered studies with external validation. These crucial steps are fundamental for clinical implementation of EVs as a source of diagnostic and predictive biomarkers in liquid biopsies from BCa patients.

## Figures and Tables

**Table 1:** Urine-based tests to aid bladder cancer clinical reasoning.

Test	Sample	Biomarker	Assay	Purpose	Sensitivity	Specificity	References
BTA TRAK^®^	Protein	Complement factor H-related	CIA	Follow-Up	0.64 (0.58–0.69)	0.77 (0.73–0.81)	[63,64,65]
BTA Stat ^®^	Protein	Complement factor H-related	SIA	Follow-Up	0.65 (0.54–0.75)	0.74 (0.64–0.82)	[64,65,66]
NMP22 BC test^®^	Protein	NMP-22	SIA	Follow-Up	0.69 (0.62–0.75)	0.77 (0.70–0.83)	[67,68]
NMP22 BladderChek test^®^	Protein	NMP-22	SIA	Diagnosis	0.47 (0.33–0.61)	0.93 (0.81–0.97)	[67,68,69]
				Follow-Up	0.70 (0.40–0.89)	0.83 (0.75–0.89)	[67,68,69]
ImmunoCyt/uCyt+^™^	Sediment	Tumor associated cellular antigens (M344; LDQ10; 19A11)	IF cytology	Diagnosis	0.85 (0.78–0.90)	0.83 (0.77–0.87)	[64,65,70]
				Follow-Up	0.75 (0.64–0.83)	0.76 (0.70–0.81)	[64,65,70]
UroVysion^™^	Sediment	Aneuploidy for chromosomes 3; 7; 17; and loss of 9p21 locus	FISH	Diagnosis	0.73 (0.50–0.88)	0.95 (0.87–0.98)	[71,72,73]
				Follow-Up	0.55 (0.36–0.72)	0.80 (0.66–0.89)	[71,72,73]
CxBladder Detect^®^	mRNA	IGFBP5; HOXA13; MDK; CDK1; CXCR2	RT-qPCR	Diagnosis	0.74 (0.65–0.81)	0.82 (0.79–0.84)	[74]
CxBladder Monitor^®^	mRNA	IGFBP5; HOXA13; MDK; CDK1; CXCR2	RT-qPCR	Follow-Up	0.91 (0.88–0.99)	0.96 (NPV)	[75]
AssureMDx^™^	DNA	FGFR3; TERT; HRAS; OTX1; ONECUT2; TWIST1	DNA methylat	Diagnosis	0.93	0.86	[76]
Xpert^®^ Bladder Cancer	mRNA	UPK1B; IGF2; CRH; ANXA10; ABL1	RT-qPCR	Follow-Up	0.84 (0.69–0.93)	0.91 (0.83–0.96)	[77]
UBC^®^	Protein	Cytokeratin 8 and 18 fragments	SIA	Diagnosis	0.61–0;65	0.77–0.82	[78]

Abbreviations: BTA; bladder tumor antigen; CIA; colorimetric immunoassay; IF; immunofluorescence; NMP; nuclear matrix protein; UBC; urinary bladder cancer antigen; FISH; fluorescence in situ hybridization; RT-qPCR; reverse transcription-quantitative polymerase chain reaction; SIA; sandwich immunoassay.

**Table 2:** Extracellular vesicles-derived biomarkers for bladder cancer.

Biomarker	Sample	EVs Separation Method	Assay	Purpose	References
EDIL-3	Protein	Sucrose cushion, ultracentrifugation	LC-MS/MS, Western blot	Diagnosis	[141]
Periostin	Protein	Ultracentrifugation	LC-MS/MS, Western blot	Prognosis	[142]
CD10, CD36, CD44, 5T4, basigin, CD73, integrin β1, integrin α6, MUC1	Protein	Sucrose cushion, ultracentrifugation	Flow cytometry, Western blot	Diagnosis	[196]
LASS2, GALNT1	mRNA	Ultracentrifugation	Microarray, RT-PCR	Diagnosis	[213]
HOTAIR, HOX-AS-2, MALAT1 OCT4, SOX2	mRNA, lncRNA	Ultracentrifugation	RT-PCR	Diagnosis	[214]
UCA1-201, UCA1-203, MALAT1, LINC00355	lncRNA	Norgen Purification Kit	RT-PCR	Diagnosis	[215]
miR-375, miR-146a	miRNA	Ultracentrifugation	Microarray, RT-PCR	Prognosis	[217]
miR-4454, miR-205-5p, miR-200c-3p, miR-200b-3p, miR-21-5p, miR-29b-3p, miR-720 /3007a	miRNA	Ultracentrifugation, Norgen Purification Kit	NanoString nCounter microRNA assay and ddPCR	Diagnosis	[218]
miR-200a-3p; miR-99a-5p; miR-141-3p; miR-205-5p	miRNA	Ultracentrifugation, Life Technologies Separation Kit	Microarray, RT-PCR	Diagnosis	[219]
miR-21-5p	miRNA	Ultracentrifugation	Microarray, RT-PCR	Diagnosis	[220]
MAGE-B4, NMP22	mRNA, protein	Norgen Purification Kit	RT-PCR	Diagnosis	[221]
MDM2, ERBB2, CCND, CCNE1, CDKN2A, PTEN, RB1	DNA	ExoQuick-TC Separation Kit	Shallow whole genome sequencing	Diagnosis	[222]
TACSTD2	Protein	Ultracentrifugation	LC-MRM/MS, ELISA	Diagnosis	[223]
Alpha-1-antitrypsin, histone H2B1K	Protein	Ultracentrifugation	Western blot, MALDI-TOF MS	Diagnosis	[224]
Resistin, GTPase NRas, EPS8L1, mucin 4, EPS8L2, retinoic acid-induced protein 3, alpha subunit of GsGTP binding protein, EH-domain-containing protein 4	Protein	Ultracentrifugation	LC-MS/MS, Western blot	Diagnosis	[225]

Abbreviations: LC-MS/MS: liquid chromatography-mass spectrometry; RT-PCR: reverse transcription polymerase chain reaction; LC-MRM/MS: liquid chromatography-multiple reaction monitoring mass spectrometry; MALDI-TOF MS: matrix assisted laser desorption ionization-time of flight mass spectrometry; mRNA: messenger RNA; miRNA: micro RNA; lncRNA: long non-coding RNA.
